# Oligodendroglioma cells synthesize the differentiation-specific linker histone H1° and release it into the extracellular environment through shed vesicles

**DOI:** 10.3892/ijo.2013.2115

**Published:** 2013-10-01

**Authors:** GABRIELLA SCHIERA, CARLO MARIA DI LIEGRO, PATRIZIA SALADINO, ROSARIO PITTI, GIOVANNI SAVETTIERI, PATRIZIA PROIA, ITALIA DI LIEGRO

**Affiliations:** 1 Dipartimento di Scienze e Tecnologie Biologiche Chimiche e Farmaceutiche (STEBICEF), Università degli Studi di Palermo, Palermo, Italy; 2 Dipartimento di Biomedicina Sperimentale e Neuroscienze Cliniche (BIONEC), Università degli Studi di Palermo, Palermo, Italy; 3 Dipartimento di Scienze Giuridiche, della Società e dello Sport, Università degli Studi di Palermo, Palermo, Italy

**Keywords:** oligodendroglioma cells, astrocytes, post-transcriptional regulation, histone variants, H1° histone, RNA-binding proteins, extracellular vesicles, shedding

## Abstract

Chromatin remodelling can be involved in some of the epigenetic modifications found in tumor cells. One of the mechanisms at the basis of chromatin dynamics is likely to be synthesis and incorporation of replacement histone variants, such as the H1° linker histone. Regulation of the expression of this protein can thus be critical in tumorigenesis. In developing brain, H1° expression is mainly regulated at the post-transcriptional level and RNA-binding proteins (RBPs) are involved. In the past, attention mainly focused on the whole brain or isolated neurons and little information is available on H1° expression in other brain cells. Even less is known relating to tumor glial cells. In this study we report that, like in maturing brain and isolated neurons, H1° synthesis sharply increases in differentiating astrocytes growing in a serum-free medium, while the corresponding mRNA decreases. Unexpectedly, in tumor glial cells both H1° RNA and protein are highly expressed, in spite of the fact that H1° is considered a differentiation-specific histone variant. Persistence of H1° mRNA in oligodendroglioma cells is accompanied by high levels of H1° RNA-binding activities which seem to be present, at least in part, also in actively proliferating, but not in differentiating, astrocytes. Finally, we report that oligodendroglioma cells, but not astrocytes, release H1° protein into the culture medium by shedding extracellular vesicles. These findings suggest that deregulation of H1° histone expression can be linked to tumorigenesis.

## Introduction

The transcriptional potential of the cell nucleus is determined by availability of transcription factors and by the structural organization of genes in the context of chromatin. Even in terminally differentiated cells, specific regions of chromatin must be remodelled to allow transcription activation or repression in response to intra- and/or extracellular signals. Linker histone H1 binds DNA in between nucleosomes and regulates chromatin higher order structures (1–5), thus also mediating differential gene expression (6,7).

The H1 family is the most divergent among histone proteins, with at least 11 different genes in humans, most of which form a cluster on chromosome 6 (chromosome 13 in mouse, and chromosome 17 in the rat) (8). In general, it is possible to distinguish between two types of H1 histone genes: clustered- and single-genes. Moreover, this peculiar distribution of H1 genes is conserved among human, mouse and rat. Each H1 protein subtype has been also suggested to have specific distribution and function in chromatin (7,9,10). In comparison with the intermediate chromatin condensing activity of H1.3, for example, other subtypes have been classified as weak condensers (H1.1 and H1.2) and strong condensers (H1.0, H1.4, H1.5, and H1.x) (10,11).

Functional differences among subtypes have been, however, difficult to identify since they probably have redundant activities in development (9,12). It is clear that linker histones are highly mobile in chromatin and that they interact with both cytosolic and nuclear proteins, thus regulating a variety of cellular processes (10); they are also able to promote epigenetic silencing of genes, by regulating both DNA methylation and histone H3 methylation (13). H1° is a linker histone subtype the expression of which has been mostly correlated with terminal differentiation (14,15).

In developing rat brain, the concentration of H1° mRNA decreases *in vivo* between the embryonal day 18 (E18) and the postnatal day 10 (P10), with inverse correlation to protein accumulation (16); the concentration of H1° mRNA also decreases in isolated neurons, between the second and the fifth day of culture in a serum-free medium, while an active synthesis of the corresponding proteins can be observed (17). The H1° gene is transcribed at the same rate at any stage studied, suggesting that it is regulated mainly at post-transcriptional level (17). Since post-transcriptional control processes are mediated by several classes of RNA-binding proteins (18–21), it was likely that developing rat brain contained mRNA-binding factors involved in H1° mRNA binding and regulation. We indeed already reported identification of a variety of H1° mRNA-binding proteins probably involved in the regulation of H1° mRNA metabolism (22–30).

A number of cell types can shed into the environment microvesicles of different sizes (MVs) under both physiological and pathological conditions (31–35). MVs contain a wide array of biological molecules, such as proteins, lipids, DNA, microRNAs and mRNAs, and have been suggested to act as means of cell-to-cell communication (36). MVs can trigger in target cells various events, including apoptosis (31,37), and cell survival and proliferation (38,39). They have also been shown to contain metalloproteinases able to digest extracellular matrix components, thus probably contributing to tissue invasion (40).

In the present study, we analyzed expression of the H1° gene in murine oligodendroglioma cells in order to shed further light on possible functions of this linker histone variant which still remains incompletely understood.

## Materials and methods

### Experimental animals

Wistar rats (Harlan, Udine, Italy) were housed in the animal house of STEBICEF Department, University of Palermo, Palermo, Italy. Procedures involving animals were in agreement with the European Community Council Directive 2010/63/EU and were approved by the University licensed veterinary. The number of animals used was minimized as much as possible.

### Cell cultures and immunofluorescence

Astrocytes were isolated from brain cortices of 2-day old newborn rats, as previously described (41), and cultured in DME/Ham’s F-12 (2/1), supplemented with 10% heat-inactivated fetal calf serum (Sigma-Aldrich, MO, USA), and 100,000 U penicillin, 100 mg streptomycin and 250 *μ*g amphotericin B (Sigma-Aldrich) per liter.

G26/24 oligodendroglioma cells were cultured in DMEMHam’s F-12 (2:1) medium supplemented with 10% fetal calf serum (FCS), and 100,000 U penicillin, 100 mg streptomycin and 250 *μ*g amphotericin B (Sigma-Aldrich) per liter, for the same time. Cell cultures were maintained in humidified 5% CO_2_/95% air, at 37°C.

Some cultures of both astrocytes and oligodendroglioma cells were then progressively adapted to a medium known as Maat-medium (MM) (41) and maintained in culture for additional 3 days, as previously described (42,43).

For immunofluorescence, cells were fixed in 96% ethanol; then astrocytes were immunostained with rabbit anti-glial fibrillary acidic protein antibodies (GFAP; Sigma-Aldrich), and oligodendroglioma cells with goat anti-actin antibodies (Santa Cruz, CA, USA). The secondary antibodies were rhodamine- or fluorescein isothiocyanate-conjugated anti-rabbit- or anti-goat immuno globulins (both from Sigma-Aldrich). Cells were finally observed under a fluorescence microscope (Olympus BX-50).

### Northern blot analysis

Northern blot analysis was performed as previously described (16). Total RNA was purified from astrocytes cultured in NIH and in MM, and from G26/24 oligodendroglioma cells, according to Chomczynski and Sacchi (44), and were separated on 1.5% agarose, 6% formaldehyde denaturing gels, transferred to nylon membranes and hybridized to a ^33^P-labeled (Perkin-Elmer, MA, USA) fragment from the plasmid pMH1° (EMBL ID: X70685), cut with EcoRI.

### Purification of total cell extracts

Cells were collected and homogenized in homogenization buffer (0.32 M sucrose; 50 mM sodium phosphate buffer, pH 6.5; 50 mM KCl, 0.5 mM spermine; 0.15 mM spermidine; 2 mM EDTA, and 0.15 mM EGTA), containing the protease inhibitors aprotinin (2 *μ*g/ml), antipain (2 *μ*g/ml), leupeptin (2 *μ*g/ml), pepstatin A (2 *μ*g/ml), benzamidine (1.0 mM), and phenylmethylsulfonyl fluoride (1.0 mM), all purchased from Sigma-Aldrich. Protein concentration was determined according to Bradford (45).

### Preparation of microvesicles from the cell culture medium

Vesicles were prepared from oligodendroglioma G26/24 and astrocyte subconfluent healthy cells grown in FCS-free medium, as previously described (33,37,40). After 24 h of culture, conditioned media were centrifuged at 2,000 × g for 10 min and then at 4,000 × g for 15 min. The supernatant was centrifuged at 105,000 × g (Ti60 Rotor, Beckman) for 90 min at 4°C. Pelleted vesicles were suspended with phosphate-buffered saline, pH 7.5 (PBS) and protein concentration in isolated vesicles was determined using Qubit^®^ Protein Assay Kit (Invitrogen, OR, USA).

### Western blot analysis

Proteins (15 *μ*g of total cell extracts) were separated by electrophoresis on denaturing 12.5% polyacrylamide slab gels (SDS-PAGE) and transferred to PVDF membrane (Immobilon P, Millipore, MA, USA), as previously described (30). Samples on the membrane were visualized by staining with Ponceau Red for 5 min. Membranes were immunostained with rabbit polyclonal anti-H1° antibodies (Santa Cruz) and mouse monoclonal anti-Hsc70 antibodies (Santa Cruz). The secondary antibodies were anti-mouse IgG (H+L), AP conjugate, and anti-rabbit IgG (Fc), AP conjugate (Promega Corporation, WI, USA).

Western blots were scanned by the ImageJ program and the values obtained were used to calculate the relative concentration of H1° in cell extracts and vesicles. The values obtained by this analysis were normalized respect to the value obtained with Hsc70 antibodies or by scanning the membrane stained with Ponceau Red. The measurements obtained from at least 3 independent experiments were finally used to calculate the relative concentrations of the analyzed proteins in the different conditions, as well as standard deviations (SD).

### Preparation of in vitro transcripts and T1 RNase protection assay

^33^P-radiolabeled H1° RNA was prepared as previously described (23), using as a template the plasmid pMH1° (46), which contains the H1° insert (EMBL ID: X70685). H1° RNA was mixed with total cell extracts (15 *μ*g), prepared as described above. For the T1 protection assay, we used a previously described method (23) except that cross-linking of RNA to proteins was performed before incubation with T1 RNase (EC 3.1.27.3; Roche, Switzerland). RNA-protein complexes were analyzed by SDS-PAGE. At the end of the run, the gel was directly exposed to X-ray film for autoradiography. The gels were also stained with Coomassie Brilliant Blue R-250 (Sigma-Aldrich), to confirm loading of equal amounts of proteins per lane.

## Results

### Expression of H1° linker histone in G26/24 oligodendroglioma cells and astrocytes

**Figure 1.**
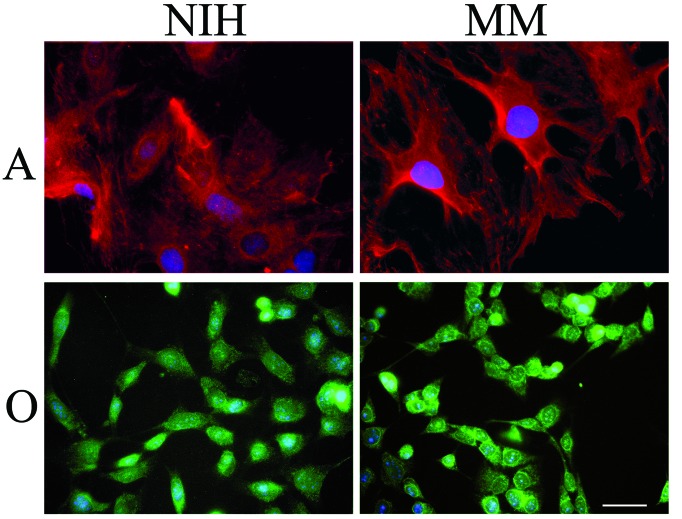
Astrocytes (A) and G26/24 oligodendroglioma cells (O), cultured in either NIH-medium (NIH) or Maat-medium (MM) for 72 h. Cells were fixed and immunostained with either anti-GFAP (A) or anti-actin antibodies (O). Bar, 20 *μ*m.

**Figure 2.**
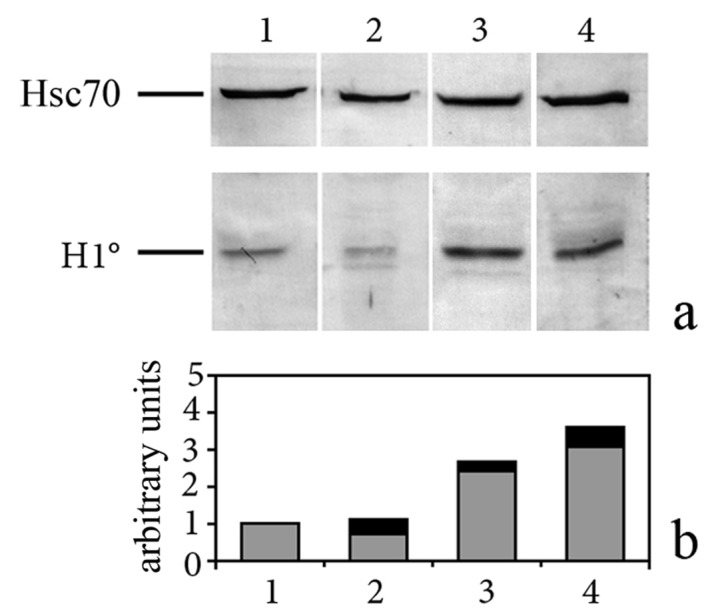
(a), Western blot analysis of total cell lysates from purified neurons (lane 1), astrocytes (lanes 2 and 3), and G26/24 oligodendroglioma cells (lane 4). Neurons were cultured in Maat-medium. Astrocytes were cultured in either NIH-medium (lane 2) or Maat-medium (lane 3). Oligodendroglioma cells were cultured in NIH-medium. Cell extracts were immunostained with anti-H1° antibodies (Santa Cruz). The upper part of the membrane was cut and immunostained with anti-Hsc70 antibodies for internal reference. (b), Graphical representation of the statistical analysis of at least three independent experiments. Grey bars indicate mean values for each condition. SDs are also indicated (black bars). Values obtained for H1° were normalized for the intensity of Hsc70 signals.

Astrocytes were cultured either in a serum-rich (NIH)- or in a serum-free medium (MM) for 72 h. As shown in Fig. 1, immunostaining of the astrocyte-specific GFAP evidenced a higher number of star-like brilliant cells when cells had been cultured in MM respect to cells cultured in NIH. Pictures of this kind suggested that astrocytes cultured in MM were a step forward, on the differentiation pathway, in respect to cells cultured in NIH. In agreement with this hypothesis, the linker histone H1°, a differentiation-specific histone variant, was expressed at higher levels in astrocytes cultured in MM (Fig. 2, lane 3) than in astrocytes cultured in NIH-medium (Fig. 2, lane 2). Concentration of H1° protein was even higher than in cortical fetal neurons, cultured in MM (Fig. 2, lane 1), already studied in the past (17). Once the relationship between H1° protein expression and differentiation was confirmed, we analyzed H1° expression in glial tumor cells. As shown in Fig. 1, there is no morphological difference between oligodendroglioma cells cultured in NIH or in MM. We found, however, that these cells (Fig. 2, lane 4) accumulate the linker histone H1° at levels comparable with those found in highly differentiated astrocytes cultured in MM (Fig. 2, lane 3). Expression of H1° histone in G26/24 cells did not change when cells were cultured in MM (data not shown).

**Figure 3.**
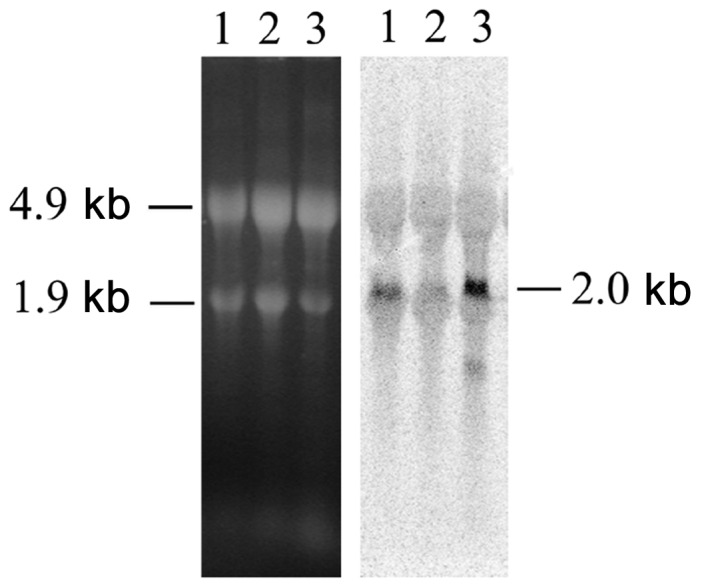
Northern blot analysis of total RNAs from astrocytes (lanes 1 and 2) and G26/24 oligodendroglioma cells (lane 3), cultured in either NIH-medium (lanes 1 and 3) or Maat-medium (lane 2) for 72 h. RNA was hybridized to a H1° probe, an EcoRI-EcoRI fragment of pMH1° which contains the entire H1° cDNA insert (EMBL ID: X70685).

Since in neurons we had found that the increase of H1° protein was accompanied by a decrease of the corresponding mRNA levels (16,17), we also investigated, by northern blot analysis, H1° RNA expression. As shown in Fig. 3, we found that the same correlation exists also in astrocytes: H1° mRNA almost disappears in differentiating astrocytes (Fig. 3, lane 2) while it is abundant in astrocytes cultured in NIH-medium (Fig. 3, lane 1). Interestingly, in G26/24 oligodendroglioma cells both H1° protein (Fig. 2, lane 4) and mRNA (Fig. 3, lane 3) are expressed at high levels.

### H1° RNA-binding proteins in G26/24 oligodendroglioma cells and astrocytes

**Figure 4.**
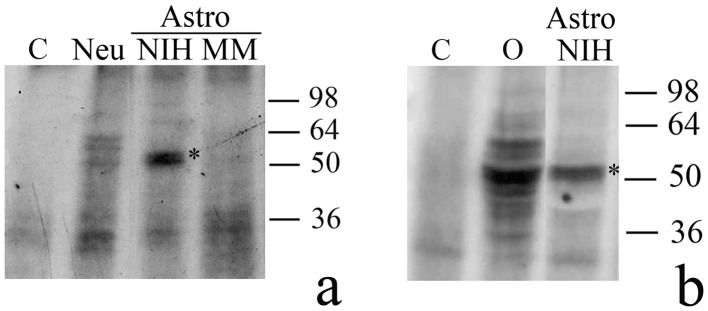
T1 RNase protection assay. H1° RNA was *in vitro* transcribed from the T3 polymerase promoter of pMH1° plasmid, purified and mixed with total cell extracts from purified neurons (a, Neu), astrocytes cultured in either NIH-medium (a and b, NIH) or Maat-medium (a, MM), or G26/24 oligodendroglioma cells cultured in NIH-medium (b, O). H1° RNA was also treated with T1 RNase in the absence of protein, as a control (C). The main bands observed at about 50 kDa are indicated by an asterisk.

The fact that the concentration of H1° mRNA decreased in astrocytes with an inverse correlation to H1° protein accumulation suggested that in these brain cells, like in neurons and whole rat brain, concentration of H1° histone was largely regulated at the post-transcriptional level. Since this level of gene expression control involves a variety of RNA-binding proteins, we tested astrocyte extracts for the presence of H1° RNA-binding factors. As shown in Fig. 4, in astrocytes cultured in the serum-rich medium (Astro, NIH) binding factors are present which forms a major complex of about 50 kDa with H1° RNA. No complex of the same apparent mass was evident in either astrocytes (Fig. 4a, Astro, MM) or neurons (Fig. 4a, Neu) cultured in MM. A major signal of about 50 kDa, and several minor ones, due to formation of H1° RNA-protein complexes, were also visible when G26/24 cell extracts were analyzed (Fig. 4b, O).

### H1° histone protein is shed by G26/24 cells through extracellular membrane vesicles

**Figure 5.**
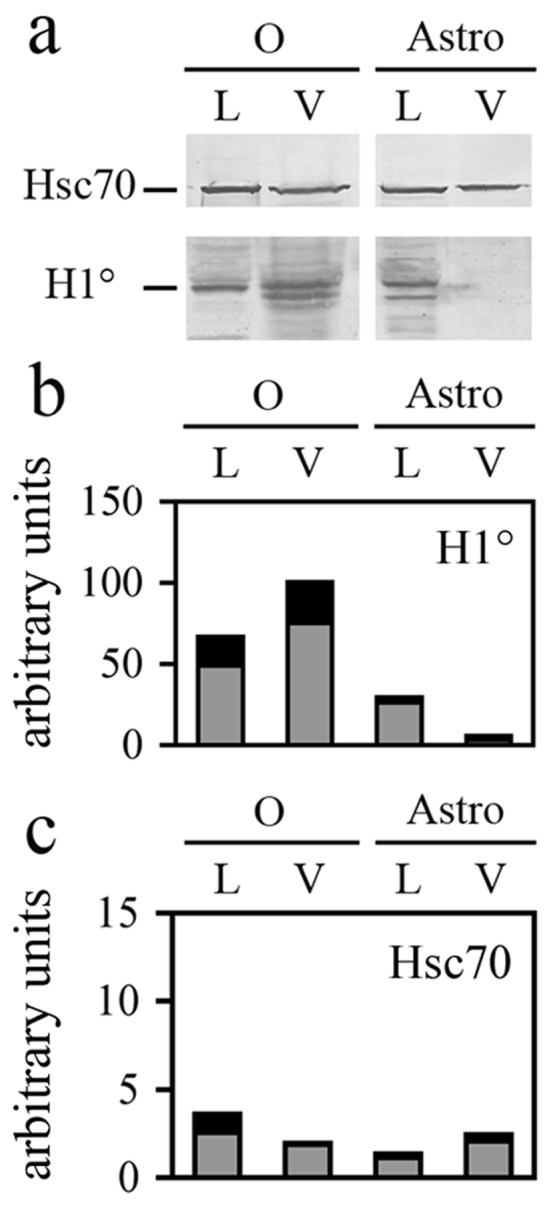
(a), Western blot analysis of total cell lysates (L) and extracellular vesicles (V) from either G26/24 oligodendroglioma cells (O) or astrocytes (Astro), immunostained with anti-H1° or anti-Hsc70 antibodies. Cells were cultured in NIH-medium. Before collecting vesicles all the cells were maintained for one night in serum-free NIH-medium. (b), Graphic representation of the statistical analysis of at least three independent experiments. Grey bars indicate mean values for each condition. SDs are also indicated (black bars). Values obtained for H1° were normalized for the intensity of Ponceau Red staining. (c), Graphic representation of the statistical analysis of at least three independent experiments. Grey bars indicate mean values for each condition. SDs are also indicated (black bars). Values obtained for Hsc70 were normalized for the intensity of Ponceau Red staining.

G26/24 cells were recently found to shed extracellular membrane vesicles which can induce neuronal death (31,37) and contain a variety of cell proteins, among which extracellular matrix metalloproteases (40). In the present study, we also tested the presence of H1° histone in the vesicles. As shown in Fig. 5a, H1° is clearly present in the vesicles shed from G26/24 tumor cells but not in those shed by astrocytes. This analysis also confirmed the already reported presence of Hsc70 in vesicles shed from oligodendroglioma cells (37). Moreover, in the present study, we report that Hsc70 is also found in vesicles released from astrocytes (Fig. 5a). The statistical analysis performed on at least three different experiments (Fig. 5b) suggested that H1° is specifically enriched in vesicles: the relative proportion of H1° in vesicles (V) shed from G26/24 (O), respect to lysates (L) of the same cells, is indeed clearly higher in comparison with the relative proportion of Hsc70 in the same samples (Fig. 5c).

## Discussion

The transcriptional potential of the cell nucleus is determined by availability of transcription factors as well as by the structural organization of genes in the context of chromatin. Even in terminally differentiated cells, specific regions of chromatin are remodelled to allow transcription activation or repression in response to intra- and/or extracellular signals. One of the mechanisms at the basis of chromatin dynamics is likely to be synthesis and incorporation of replacement histone variants, such as the core H3.3 histone and the linker H1° histone. In developing brain, as well as in fetal neurons differentiating in culture, H1° mRNA is progressively down-regulated *in vivo* at the time of rat brain maturation (16,17), with an inverse correlation to synthesis and accumulation of H1° protein, although the transcriptional activity of H1° gene is unaffected by terminal differentiation. This finding suggested that H1° expression in the brain was mostly regulated at the post-transcriptional level (17).

As shown in the present study, like in the whole brain and in isolated neurons, the linker histone H1° is expressed at higher levels in astrocytes cultured in a serum-free medium (MM) in which they acquire a clearly differentiated star-like appearance. In these cells, concentration of H1° protein is even higher than in cortical fetal neurons, cultured in the same medium. Moreover, like in neurons, H1° mRNA almost disappears with protein accumulation. It is likely that H1° mRNA is destabilized and degraded at higher rates concomitant with its increased engagement with the translational apparatus.

How can the availability of H1° mRNA to the ribosomes be controlled? We already knew that a variety of H1° mRNA-binding factors exist in the rat brain (22–30). Now we evidenced an RNA-protein covalent complex of about 50 kDa, which disappears when astrocytes are cultured in differentiating conditions. Since the T1 RNase assay is a functional test, we cannot say whether the factor disappears during differentiation or undergoes a post-translational modification that abolishes its binding activity.

Unexpectedly, in glial tumor cells concentration of both H1° mRNA and protein is very high and does not correlate with a decrease of proliferation rate. Thus the still unknown mechanism responsible for the inverse correlation between H1° mRNA and protein concentrations does not work in oligodendroglioma cells. Complexes with a size similar to the complex seen in undifferentiated astrocytes do form, but they are probably not able to block access to ribosomes. Most importantly, synthesis of a high level of H1° histone protein does not correlate with a decrease of proliferation rate.

Since we already knew that G26/24 cells actively shed extracellular microvesicles (MVs), which contain a variety of proteins (31,37,40), we asked whether oligodendroglioma cells are able to unload H1° into the extracellular environment. Here we report that indeed H1° histone is present in MVs shed by oligodendroglioma cells but not in those shed by astrocytes, even if both populations of MVs contain, for example, Hsc70 chaperone. Although the role of shedding in tumor cells is not yet completely understood, it could be also involved in eliminating proteins from cells (such as the H1° histone) that could be otherwise able to counteract proliferation.
